# Population Structure of an Invasive Parthenogenetic Gastropod in Coastal Lakes and Estuaries of Northern KwaZulu-Natal, South Africa

**DOI:** 10.1371/journal.pone.0024337

**Published:** 2011-08-31

**Authors:** Nelson A. F. Miranda, Renzo Perissinotto, Christopher C. Appleton

**Affiliations:** School of Biological and Conservation Sciences, University of KwaZulu-Natal, Westville Campus, Durban, South Africa; Institut de Biologia Evolutiva - Universitat Pompeu Fabra, Spain

## Abstract

**Background:**

Estuaries and coastal lakes receive little attention despite being heavily invaded by non-indigenous invasive species (NIS). In these situations, studies of population dynamics in invaded habitats can provide valuable insights into how NIS interact with new environments. *Tarebia granifera* is a prosobranch gastropod from south-east Asia which has invaded other sub-tropical parts of the world. This study addresses whether a small number of key environmental factors influences gastropod communities, and specifically how the population density and size structure of *T. granifera* were influenced by environmental change in estuaries and coastal lakes in southern Africa.

**Methodology/Principal Findings:**

*T. granifera*'s density, number of brooded juveniles and size structure were measured at the St. Lucia Estuary, Mgobozeleni Estuary, Lake Sibaya and Lake Nhlange. Size structure was classified according to shell height (SH). All dissected individuals were found to be female and free from trematode infection. Salinity, water depth, temperature, and pH were the main factors correlated with population density of gastropod communities. *T. granifera* often reached densities well over 1000 ind. m^−2^, displacing indigenous gastropods and becoming a dominant component of the benthic community. *T. granifera* successfully invaded estuaries despite frequent exposure to high salinity and desiccation, which could together eliminate >97% of the population. The persistence of *T. granifera* was ensured due to its high fecundity and the environmental tolerance of large adults (20–30 mm SH) which carried an average of 158±12.8 SD brooded juveniles. Repeat introductions were not essential for the success of this parthenogenetic NIS.

**Conclusion/Significance:**

There is a need for a broader study on the reproductive biology of *T. granifera* (including the previously overlooked “brood pouch ecology”), which affects population dynamics and may be relevant to other parthenogenetic NIS, such as *Melanoides tuberculata* and *Potamopyrgus antipodarum*.

## Introduction

Although asexual reproduction can be found in numerous organisms, it is rare to find obligate parthenogenetic taxa, in which single organisms can only reproduce by producing genetically identical offspring [1]. Some gastropods show patterns of geographic parthenogenesis, where asexual populations occupy different habitats to populations that reproduce sexually [2], [3]. Parthenogenetic populations may have several advantages for establishment in new and variable habitats: a single parthenogenetic organism can start a population [3]–[6]; the genotype is isolated from gene flow and adaptations to the new habitat, especially in “general purpose genotypes”, can thus not be broken by recombination (see frozen - niche variation [7]); in habitats where populations undergo frequent local extinction and recolonization events, genetic bottleneck and drift effects will have less negative fitness consequences for asexual populations [8]; also, according to the Red Queen Hypothesis, reduced biotic interactions with parasites and predators favor asexual populations (see [9], but also [10]).

The geographic patterns and rates of species' invasion are changing on an unprecedented scale due to direct and indirect anthropogenic action [11]. Non-indigenous invasive species (NIS) are a serious threat to biodiversity [12], particularly in estuarine and coastal environments [13], [14]. Fortunately, relatively few introduced species are successful in establishing populations, fewer go on to spread and fewer still become pests [15]. Chance plays a role in the invasion success of all NIS and there can be many repeated attempts before an invasion is successful [12], [16]. However, certain biological and ecological characteristics are thought to increase the probability of invasion [17]. In this context, it is not surprising that an introduced parthenogenetic species pre-adapted to colonize marginal habitats with wide physiological tolerance, high fecundity and very high population densities would also make a successful NIS [18].

*Tarebia granifera* is a prosobranch gastropod (Thiaridae) originally from south-east Asia. This parthenogenetic species has a brood pouch and gives birth to fully developed juveniles. *T. granifera* has high fecundity and has been reported to reach densities over 20 000 ind. m^−2^ [19]. It has invaded several sub-tropical parts of the world, including Texas, Hawaii, Caribbean islands, Mexico and Israel [20]–[23]. In South Africa, *T. granifera* has invaded an increasing number of estuaries and coastal lakes over the past decade [19]. The species is regarded as a freshwater dweller, but its recent invasion patterns [19], a physiological tolerance study [24] and a strontium isotope (^87^Sr/^86^Sr) study of fossils dating back 1.5 million years [25] suggest that this species is pre-adapted to brackish environments. The shallow marginal habitats of coastal lakes and estuaries can be extremely variable environments [26], [27]. Stochastic events involving changes in water level and salinity have been observed to repeatedly wipe out most of the *T. granifera* population and yet, this NIS not only persists but often becomes a dominant component of the shallow-water benthos (pers. obs.).

This study aimed to address two questions: 1) is gastropod community structure influenced by a small number of key environmental factors? 2) What are the longer term effects of environmental change on *T. granifera* within a variable estuarine setting? The size structure of *T. granifera* (in terms of shell height size classes) has previously only been described for freshwater bodies and in the laboratory. Differences in population density and size structure over time and under different environmental conditions revealed how *T. granifera* populations persisted during unfavorable periods and then recovered. *T. granifera*'s reproductive output was tentatively assessed in terms of number of unborn juveniles in the brood pouch.

## Materials and Methods

### Ethics Statement

Permission for this study was granted under a Research Agreement with the iSimangaliso Wetland Park Authority for the project titled “Climate Change and the Management of KZN estuaries: St Lucia Estuary”.

### Study site

**Figure 1:**
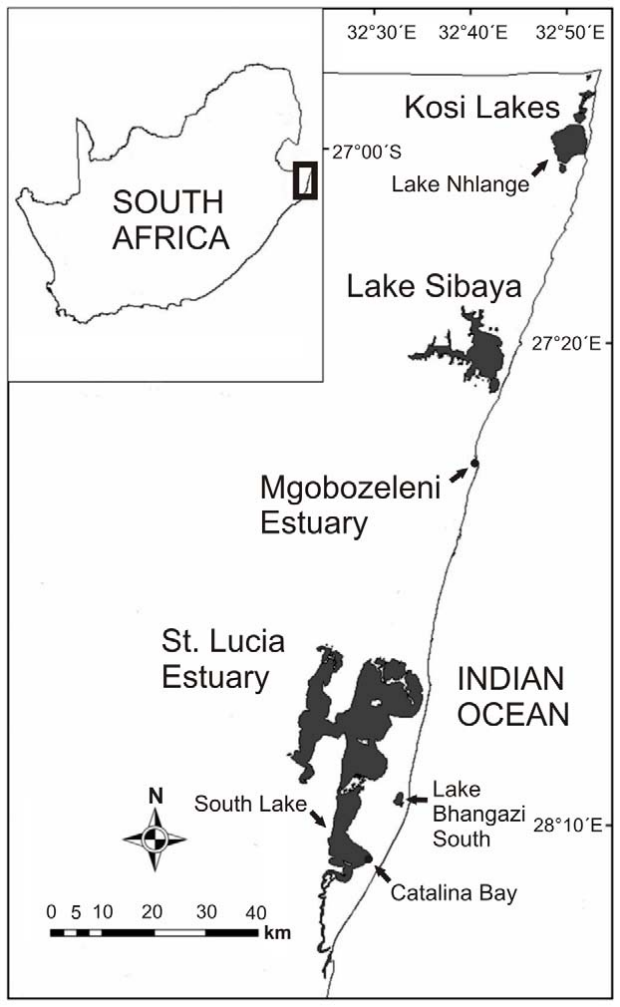
Map of Maputaland. The Kosi Lakes, Lake Sibaya and the St. Lucia Estuary are Ramsar Wetlands of International Importance within the iSimangaliso Wetland Park, a UNESCO World Heritage Site in northern KwaZulu-Natal, South Africa.

**Figure 2:**
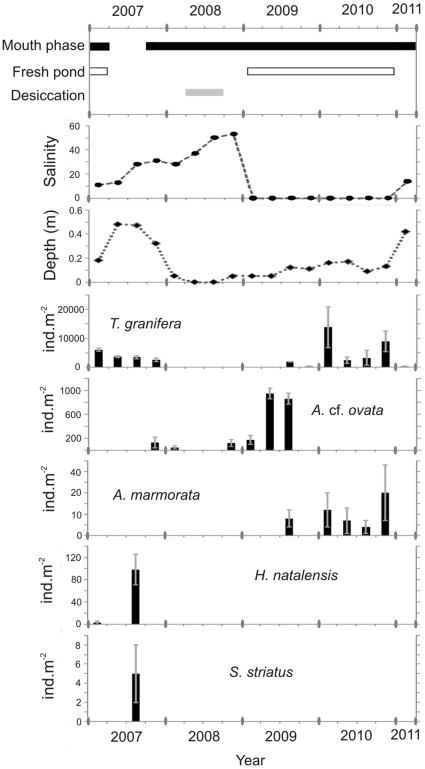
Gastropod population densities (± SD) under changing salinities and depths. Data were collected at Catalina Bay, in the St. Lucia Estuary from 2007 to 2011 at quarterly intervals. The black horizontal bar represents a closed mouth phase, the white bar represents the presence of a freshwater pond, and the grey bar represents the desiccation of most of the sampling area. Species: *Tarebia granifera*, *Assiminea* cf. ovata, *Aplexa marmorata*, *Haminoea natalensis* and *Stylocheilus striatus*.

The St. Lucia Estuary is the largest estuarine lake in Africa, with a surface area of ≈325 km^2^ and average depth of 0.9 m [28]. Recently, this estuary has been experiencing unprecedented low water levels and the mouth has been closed for the most part from 2002 to present. There is a reversed salinity gradient and salinities over five times higher than seawater were recorded in the most northerly parts of the system [29]. However, areas such as the eastern shores of South Lake, receive a considerable input of freshwater from sand dune aquifers [30]. Samples were collected at Catalina Bay (28°13′S, 32°29′E), on the eastern shores of South Lake (Fig. 1). In March 2007, the St. Lucia Estuary mouth breached and seawater from the Indian Ocean entered the system [29], increasing water levels and introducing a number of marine species, including the sea hare *Stylocheilus striatus* (Fig. 2).

Mgobozeleni Estuary (27°32′S, 32°40′E; Fig. 1) has a surface area of 0.014 km^2^, maximum width of 25 m and average depth of 0.3 m [31]. This estuary is supplied with freshwater from Lake Mgobozeleni and is strongly influenced by tidal regimes [32]. It is a typical Temporarily Open/Closed Estuary (TOCE, Perissinotto et al. 2010) and a combination of low rainfall and spring tide may result in the periodical closure of the mouth [33].

Lake Sibaya has a surface area of 60 to 77 km^2^ and an average depth of 13 m [34]. This land-locked freshwater lake currently undergoes wide fluctuations in water level [34]. Samples were collected on the eastern shores (27°22′S, 32°42′E; Fig. 1).

Lake Nhlange (26°57′S, 32°49′E) is the largest of the Kosi Lakes (Fig. 1). The surface area of this lake varies from 30.7 to 37 km^2^ and the average depth is 7.2 m [35]. Lake Nhlange is connected to the ocean via channels and other lakes but its salinity is low [35]. Samples were collected on the western shores.

### Sampling procedure

The St. Lucia Estuary was surveyed at quarterly intervals between February 2007 and March 2011; Mgobozeleni Estuary, Lake Sibaya and Lake Nhlange were surveyed during the wet seasons in 2009 and 2010 (Table S1).

### Physical and chemical parameters

All samples were taken in shallow marginal habitats (<2 m depth). Salinity, dissolved oxygen, pH and temperature were measured with a YSI 6920 multiprobe. Sub-surface water samples were sieved through a GF/F filter and the supernatant was analysed for nitrates and phosphates with a Skalar SAN^++^ continuous flow nutrient analyzer. Sediment samples were dried and weighed before being sieved through a 2000 µm sieve. The finer sediment was analysed by a Malvern Analyser. The median sediment particle size was then calculated by taking into account the weighted sediment retained on the sieve.

### Gastropods

Triplicate macrofauna samples were taken with a Zabalocki-type Ekman grab (area = 0.0236 m^2^). Immediately after collection, samples were washed through a 500 µm sieve and the material retained was preserved in 5% formaldehyde solution. In the laboratory, gastropods were sorted from each sample and counted in order to determine density (ind. m^2^). *Tarebia granifera* shell height (SH) was measured with a Vernier caliper to the nearest 0.01 mm. Each specimen was inspected for shell damage and if the first whorls were missing, its (damaged) shell height was multiplied by the ratio of average SH/average height of the last whorl of unaffected (undamaged) specimens belonging to that population and appropriate size class [36]. Thus, the corrected SH was used in spatial and temporal comparisons. The gastropods were sorted into one of the following 10 size classes according to (corrected) SH: <1 mm; 1–5.99 mm; 6–7.99 mm; 8–9.99 mm; 10–11.99 mm; 12–13.99 mm; 14–15.99 mm; 16–17.99 mm; 18–19.99 mm; 20–30 mm. No less than five individuals belonging to each size class were dissected. The gonads were inspected to determine sex and presence of trematode parasites. The brood pouch of adult specimens (6–30 mm SH) was carefully dissected and the shelled juveniles contained inside were counted.

### Data analysis

Analysis of covariance (ANCOVA) using size class as a covariate was used to assess differences in average number of unborn juveniles per brood pouch between sampling events, and between locations. A two-way analysis of variance (ANOVA) was used to determine if *T. granifera* densities differed temporally (in terms of year×season) at Catalina Bay, St. Lucia Estuary. An ANOVA and Tukey's Honestly Significant Difference (HSD) multiple comparisons were conducted to determine if *T. granifera* densities differed between locations. The relationship between shell height and number of juveniles in brood pouch was analysed with Pearson's correlation. All data were log-transformed to meet normality requirements. The statistical package PASW version 18 for Windows was used.

**Figure 3:**
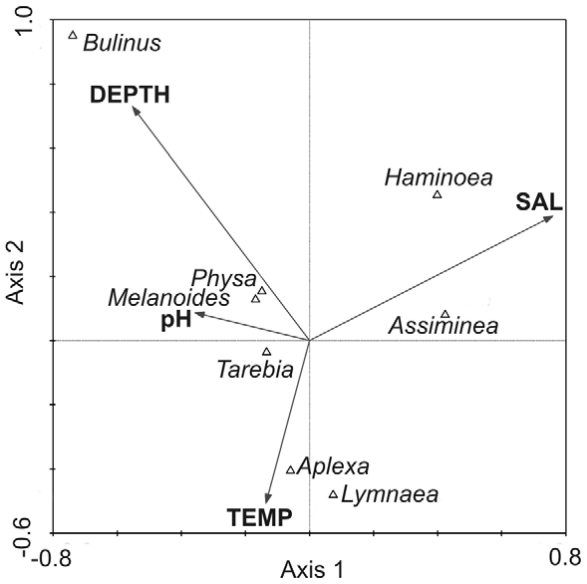
Maputaland gastropod population densities correlated with physical and chemical parameters. First and second axes of a canonical correspondence analysis performed on gastropod population densities and physical and chemical parameters measured at Lake Nhlange, Lake Sibaya, Mgobozeleni Estuary and St. Lucia Estuary in northern KwaZulu-Natal, South Africa. Data were collected from 2007 to 2011at quarterly intervals. Physical and chemical parameter vectors: salinity (SAL), depth, water temperature (TEMP) and pH. Species: *Tarebia granifera* (*Tarebia*), *Melanoides tuberculata* (*Melanoides*), *Physa acuta* (*Physa*), Assiminea cf. *ovata* (*Assiminea*), *Haminoea natalensis* (*Haminoea*), *Aplexa marmorata* (*Aplexa*), *Lymnaea natalensis* (*Lymnaea*) and *Bulinus natalensis* (*Bulinus*).

A canonical correspondence analysis was conducted between log-transformed gastropod (8 species, Fig. 3) abundance data and standardized environmental data (depth, salinity, temperature, dissolved oxygen, pH, median sediment particle size, turbidity, nitrates and phosphates) collected at the study site between 2007 and 2010. A forward selection model was used to determine the four environmental variables which best explained the variation in density data of gastropod species. The CANOCO software version 4.5 was used for this purpose.

## Results

### Canonical correspondence analysis

The canonical correspondence analysis showed that the combination of salinity, depth, temperature and pH explains 78% of the variation in the abundance data of gastropod species (Table 1). The first two axes together explain 58.3% of total variability in species abundance data (Table 1).

**Table 1:** Canonical correspondence analysis performed on abundances of Maputaland gastropods and physical and chemical parameters.

		Axis 1	Axis 2	Axis 3
**Eigenvalues**		0.392	0.191	0.146
**Cumulative percentage:**	**of species data**	20.1	29.9	37.3
	**species-environment relation**	50.3	74.8	93.5
	**Species-environment correlation**	0.82	0.75	0.656
**Component loadings:**	**Depth**	−0.628	0.737	−0.223
	**Salinity**	0.803	0.384	−0.298
	**Temperature**	−0.190	−0.501	−0.262
	**pH**	−0.343	0.085	0.636

### Population densities

In the St. Lucia Estuary, an increase in salinity was associated with increases in density of indigenous gastropods such as *Haminoea natalensis* and *Assiminea* cf. *ovata* and the decline of *Tarebia granifera* (Fig. 2). The mouth was only open for 6 months, and in 2008 water levels dropped dramatically, leaving vast areas dry. In 2009, freshwater pooled on the eastern shores of the South Lake where non-indigenous *T. granifera* as well as *Aplexa marmorata* were found (Fig. 2).

*T. granifera* population density was not significantly different in terms of year×season at Catalina Bay (Two-way ANOVA: *F*_2113_ = 1.051, *P*>0.05). *T. granifera* density was however significantly different between locations (Catalina Bay, Mgobozeleni Estuary, Lake Sibaya and Lake Nhlange, 2007–2010 wet seasons only) (ANOVA: *F*_3,31_ = 30.359, *P*<0.05). *T. granifera* densities at the freshwater Sibaya and Nhlange lakes were significantly different from densities at the brackish St. Lucia and Mgobozeleni Estuaries (Tukey's HSD: *P*<0.05).

### Size classes and juveniles in brood pouch

All *T. granifera* found in this study were female. An inspection of the gonads and digestive gland also revealed no trematode infections. Specimens with shell height smaller than 6 mm had under-developed brood pouches, which did not contain shelled juveniles. Variable sizes of juveniles were found within the brood pouch.

The shells of *T. granifera* were most severely damaged at Catalina Bay, where in 2009 and 2010 on average 78% of the population showed signs of shell erosion and SH was reduced by an average of 12.3%. The damage was concentrated in the size classes 6–13.99 mm SH.

**Figure 4:**
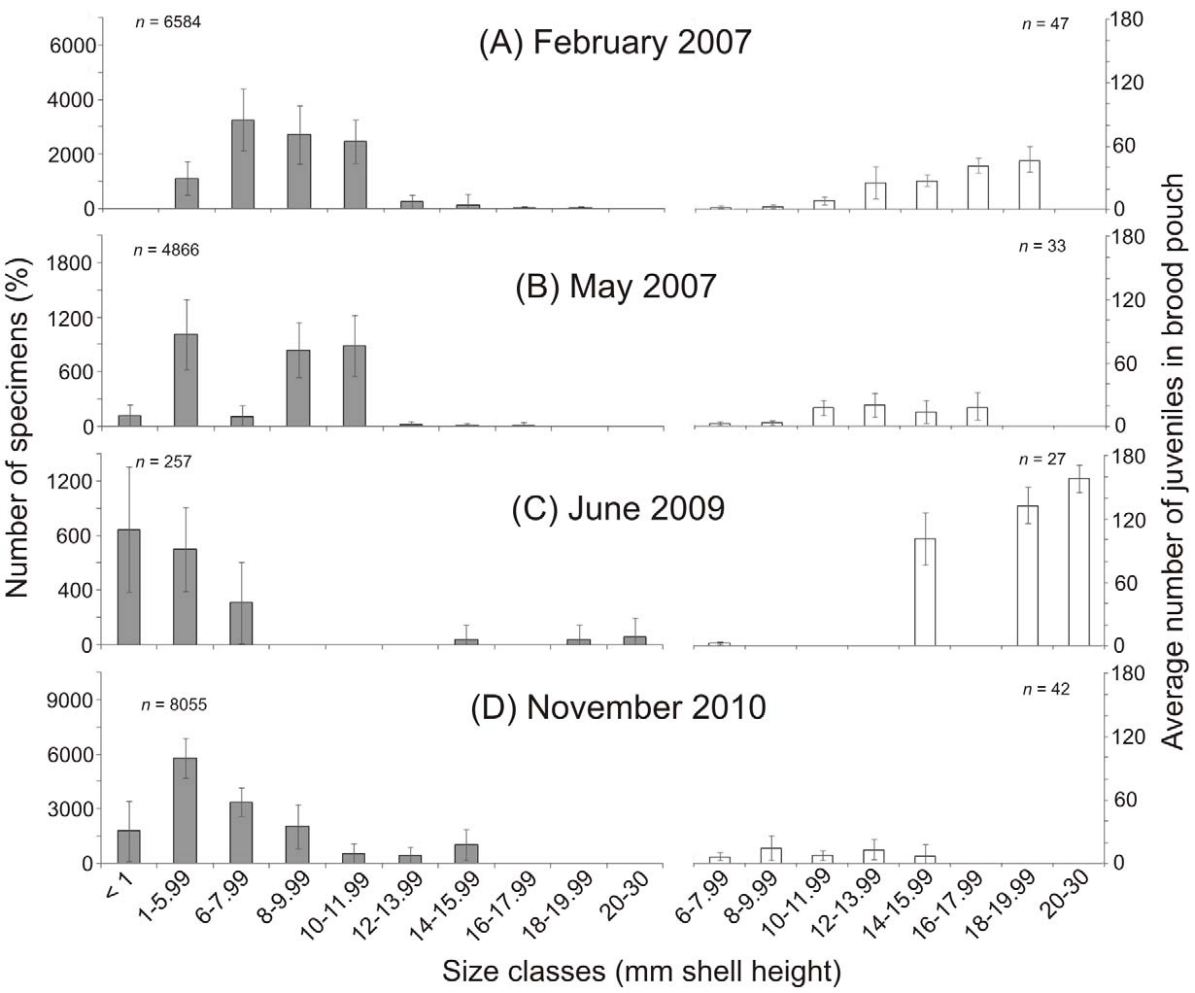
*Tarebia granifera* size structure over time. Size class (mm shell height) distribution of *T. granifera* specimens (percentages ± SD) and average number of unborn juveniles (± SD) per adult size classes (6–30 mm shell height) collected by triplicate Ekman grab at Catalina Bay, in the St. Lucia Estuary during (A) February 2007, (B) May 2007, (C) June 2009 and (D) November 2010.

At Catalina Bay in February 2007, the distribution of the *T. granifera* population was unimodal, with size class 6–7.99 mm SH being the most common and contributing 32.7%±11 SD of the total (Fig. 4A). Each adult of 6–7.99 mm SH carried on average 1.3±0.6 SD juveniles, whereas each adult of 18–19.99 mm SH carried 48.6±12.1 SD juveniles in its brood pouch (Fig. 4a). In May 2007, after mouth breach and under salinity≈28 (Fig. 2, Table S1), the *T. granifera* population appeared to have a bimodal distribution and the size class 6–7.99 mm SH was poorly represented (Fig. 4B). The largest size class represented was 16–17.9 mm SH and each snail in this class carried an average of 18±13.4 SD juveniles in its brood pouch. In 2008 there were no *T. granifera* recorded at the seepage area of Catalina Bay, which dried out completely resulting in the *T. granifera* population being reduced by >97%. In June 2009, seepage water accumulated to form a freshwater pond independent of the South Lake. Large *T. granifera* (14–30 mm SH) were found making conspicuous trails on the sediment surface and the largest (20–30 mm SH) carried on average 158±12.8 SD juveniles in the brood pouch (Fig. 4C). 77.3%±22.8 SD of the total population was composed of juveniles (Fig. 4C, Table S1). In November 2010, 50.5%±18.1 SD of the total population was composed of juveniles at Catalina Bay (Fig. 4D).

**Figure 5:**
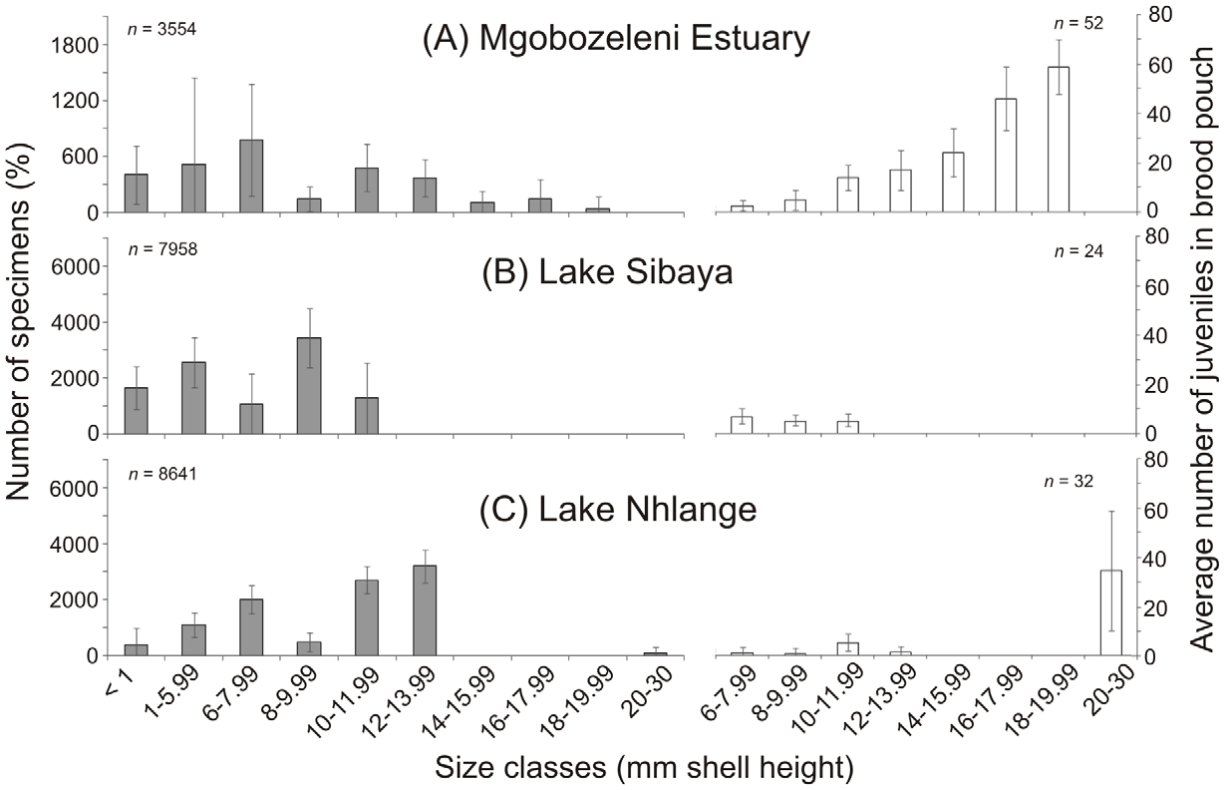
*Tarebia granifera* size structure at different locations. Size class (mm shell height) distribution of *T. granifera* specimens (percentages ± SD) and average numbers of unborn shelled juveniles (± SD) per adult size class (6–30 mm shell height) collected by triplicate Ekman grab in November 2010 at (A), Mgobozeleni Estuary (B) Lake Sibaya and (C) Lake Nhlange.

At the Mgobozeleni Estuary, juveniles contributed 30.9%±40.3 SD and adults ranged from 6 to 19.99 mm SH (Fig. 5A). At Lake Sibaya, the largest adults found on the open terraces of the eastern shores were 10–11.9 mm SH and carried on average 4±1.3 SD juveniles in the brood pouch (Fig. 5B). At Lake Nhlange, 59%±10.3 SD of the population was composed of size classes 10–13.99 mm SH in November 2010 (Fig. 5C). The largest adults (20–30 mm SH) carried 35±24 SD juveniles in the brood pouch (Fig. 5C).

*T. granifera* shell height and number of unborn juveniles in brood pouch were positively correlated (Pearson's correlation coefficient: *r* = 0.806, *P*<0.01, *n* = 186). There was no significant difference in average number of unborn juveniles per brood pouch between sampling events at Catalina Bay (ANCOVA: *F*_11,59_ = 1.626, *P* = 0.115). There was also no significant difference in average number of unborn juveniles per brood pouch between locations (ANCOVA: *F*_3,57_ = 1.174, *P* = 0.328).

## Discussion

*Tarebia granifera population* densities were often well over 1000 ind. m^−2^ regardless of location, making this NIS a dominant component of the local invertebrate macrofauna. However, at the St. Lucia and Mgobozeleni estuaries, population densities were variable, whereas at the Sibaya and Nhlange coastal lakes the *T. granifera* populations appeared relatively stable (Table S1). South African estuaries are particularly variable environments in terms of salinity and water depth and these factors can change unpredictably and directly influence macrofauna [27]. As expected for any benthic invertebrate, the spatial distribution of *T. granifera* was heterogeneous [37], [38], [39]. In general, shallow water (depth<2 m) freshwater sources and sheltered bays with organic deposits seemed to be favored. Extreme changes in salinity and water depth, which affected the populations of all gastropod species, were particularly evident at the St. Lucia Estuary during the study period (Fig. 2). Yet the highest *T. granifera* population densities were recorded at Catalina Bay (Table S1). Indeed, it has been suggested that the high densities of *T. granifera* may minimize the risk of extirpation under harsh conditions [10].

Salinity, water depth, temperature and pH were identified as the four main factors associated with the population density of the dominant gastropod species currently found in the estuaries and coastal lakes of Maputaland (Fig. 3, Table 1). Most gastropods appear to be associated with a specific set of environmental conditions. For instance, *Bulinus natalensis* was associated with deeper water, whereas *Aplexa marmorata* and *Lymnaea natalensis* were associated with very shallow and warm waters (Fig. 3). *Haminoea natalensis* and *Assiminea* cf. *ovata* were the only species associated with high salinity (Fig. 3). However, in comparison to other gastropods, *T. granifera* tended to be least associated with any one environmental factor (Fig. 3) and was the most widespread and abundant gastropod in all study areas.

The *T. granifera* population density did not appear to undergo seasonal patterns and year-round births were recorded. The average number of juveniles per brood pouch, which was assumed to indicate reproductive output, did appear to increase when the salinity suddenly increased in the St. Lucia Estuary in 2007 and also when the population was recovering in 2009 (Table S1). An increase in average number of juveniles per brood pouch was also measured during *T. granifera*'s recovery at the Mgobozeleni Estuary. *T. granifera* may increase its reproductive output in response to disturbances, such as sudden salinity increases that negatively affects the population, therefore accelerating its recovery. However, size classes were not taken into account in this interpretation.

The structure of the *T. granifera* population was defined in terms of its proportional contribution to different shell height size classes. *T. granifera*'s shell height (SH) ranged from >1 mm to 28.75 mm. The largest specimens were recorded at the St. Lucia Estuary. *T. granifera* adults collected in Lake Nhlange and particularly at Lake Sibaya tended to be small (Fig. 5B), although larger specimens were found in sheltered and eutrophic bays. The size of specimens may have been influenced by food availability and quality [36], [40]. Both Lake Nhlange and Sibaya are nutrient poor and other species have been reported to have unusually smaller sizes [41]. In contrast, the St. Lucia Estuary has been reported to have very high levels of accumulated microphytobenthos that *T. granifera* can feed on [42], [43]. *T. granifera* maturity in this study was reached between 6 and 7.99 mm SH [36]. The erosion of *T. granifera* shells at Catalina Bay was most likely caused by low pH and abrasive effect of sand particles. Interstitial pore water at Catalina Bay, which seeped from sand dune aquifers, had a pH≈6.3. *T. granifera* populations are sensitive to low pH [36], [44]. Lake Bhangazi South [45] (Fig. 1) and surrounding small streams tend to have low pH and this may partially explain why the *T. granifera* invasion has not taken hold in those habitats (pers. obs.).

*T. granifera* size classes were clearly affected by stochastic events in different ways. Size classes between >1 and 7.99 mm SH seemed particularly vulnerable to the sudden increase in salinity during the 2007 mouth breach of St. Lucia (Fig. 4A–B). Yet a higher proportion of juveniles was recorded during that period (Fig. 4A–B) and the juvenile to adult ratio also increased (Table S1), indicating that birth rate did not slow down. The ensuing desiccation in 2008 killed most of the population, with only the largest adults able to survive such extreme conditions. This was at least in part due to their tolerance to desiccation [46], ability to burrow and undergo periods of quiescence [20], but also because large *T. granifera* probably took shelter in freshwater seeps. In comparison, smaller and juvenile snails are less tolerant to desiccation [46] and also move over smaller spatial ranges [37], therefore they were not able to reach freshwater seeps and survive. Once favourable conditions were re-established in 2009, adults returned and gave birth to large numbers of juveniles, thus quickly increasing the juvenile to adult ratio. These larger adults carried a great number of juveniles in their brood pouch, thus contributing to the perceived high reproductive output during the recovery period of 2009 (Fig. 4C, Table S1). By the end of 2010, the Catalina Bay population had recovered and its structure and density was comparable to those of other *T. granifera* populations in Maputaland.

The evolutionary significance of brooding may be unclear [47]. However, in *T. granifera*, brooding is associated with increased parental care, which minimizes mortality of vulnerable early life stages. The anatomy of the brood pouch of *T. granifera* is similar to that of *M. tuberculata* [36], [48] and the larger the animal, the greater the number of juveniles it can carry. The current study also found a variety of sizes of juveniles in the brood pouch. This suggests that juveniles may be retained during adverse conditions and raises the question of whether the number of juveniles present in a brood pouch is an adequate indicator of fecundity. Under salinities higher than 20, an adult can shift its energy from reproduction to survival [49], [50], which involves entering a quiescent state [24]. Therefore, an increase in number of juveniles in brood immediately after an increase in salinity may be due to retention, rather than increase in reproduction rate. The increase in the ratio of juveniles to adults during adverse conditions could then be explained by the release of brooded juveniles after the death of adults and/or births by large and more tolerant adults. An empirical study is needed to address this hypothesis since many factors affect birth [20].

Propagule pressure can be described in terms of quantity of released propagules, the quality of the propagules and the quantity of release events [51]. *T. granifera* reaches high population densities and a single adult with several brooded juveniles may start a new population. The brood pouch plays an important role in the internal cycle of propagule production and dispersal [11], which ensures *T. granifera* persistence and spread even if there is no further input from an external propagule pool. Juveniles grow within the protection of the brood pouch, thus increasing their chance of survival and reducing the time to maturity after birth. This may ensure year-round continuity in *T. granifera* reproductive output, despite unfavorable and unpredictable events.

*T. granifera* is known to displace indigenous gastropods in freshwater [19], [22], [23] but its ecological impacts in brackish water are largely unknown. However, ecological impact on estuaries and coastal lakes is difficult to assess because of unprecedented overlaps and interactions between NIS and other stressors [52] such as drought intensification. It would have been very useful to make a comparison of *T. granifera* size structure with that of indigenous *Melanoides tuberculata* and *Bellamya capillata* at Lake Sibaya, since all three species reproduce via parthenogenesis [53]. Unfortunately though, the historically abundant native snails were not found during this study, having possibly been displaced by *T. granifera*. However, it is likely that they persist in deeper water or in parts of Lake Sibaya that were not surveyed. *T. granifera* has been reported to displace *M. tuberculata* under natural settings [23], [54]. However, at Lake Nhlange, *T. granifera* and *M. tuberculata* have been found together in a small sheltered and eutrophic bay, but *T. granifera* was more numerous.

An understanding of the population dynamics of NIS is important for predicting their interaction with the environment and determining the best control strategy [55]. Already invaded habitats with reduced biotic influences can present an opportunity to gain insights into how NIS interact with the environment. This study has revealed how *T. granifera*'s population density and structure can change in variable estuarine and coastal environments. High densities, fecundity, and particularly the environmental tolerance of adults with brooded juveniles, ensure the persistence of *T. granifera* despite frequent mouth breaching events and desiccation. The mode of reproduction and the type of embryo development affect population dynamics. However, the ecology of the post-larval and pre-birth stage in the life history of *T. granifera*, which takes place within its brood pouch, needs further consideration. It is suggested that this brood pouch ecology plays a very significant role in the establishment and spread of *T. granifera*, thus also affecting population dynamics. These findings are also relevant to invasions by other parthenogenetic NIS, such as *M. tuberculata* and *P. antipodarum*, across the world.

## Supporting Information

Table S1 **Summary of physical and chemical parameters, and** ***Tarebia granifera*** **populations in Maputaland estuaries and coastal lakes.**
(DOC)
