# Supplementary material for: Population Structure of an Invasive Parthenogenetic Gastropod in Coastal Lakes and Estuaries of Northern KwaZulu-Natal, South Africa
Source: PLoS One. 2011 Aug 31;6(8):e24337. doi: 10.1371/journal.pone.0024337 (PMC3164166; doi:10.1371/journal.pone.0024337)
Supplement: Table S1 — Summary of physical and chemical parameters, and Tarebia granifera populations in Maputaland estuaries and coastal lakes. (DOC) [file pone.0024337.s001.doc]

**Table S1.** Summary of physical and chemical parameters, and *Tarebia granifera* populations in Maputaland estuaries and coastal lakes.

| **Location** | **Date and {season}** | **Depth (m)** | **Salinity** | **Temperature (°C)** | **pH** | **Density (Ind.m^-2^)** | **Juv. : Adults (%)^a^** | **Juv. in brood / Adults ^b^** |
| --- | --- | --- | --- | --- | --- | --- | --- | --- |
| Catalina Bay | Nov 2010 **{**wet**}** | 0.10 ± 0.05 | 0.82 ± 0.20 | 28.21 ± 0.34 | 7.89 ± 0.98 | 8874 ± 569 | 50.5 : 49.5 | 6.2 |
| Catalina Bay | Jun 2009 **{**dry**}** | 0.05 ± 0.01 | 0.46 ± 0.31 | 26.44 ± 0.16 | 8.61 ± 0.18 | 1899 ± 121 | 77.3 : 22.7 | 44.4 |
| Catalina Bay | May 2007 **{**dry**}** | 0.10 ± 0.12 | 28.7 ± 0.45 | 25.45 ± 0.50 | 7.85 ± 0.66 | 3451 ± 352 | 37.6 : 62.4 | 9.2 |
| Catalina Bay | Feb 2007 **{**wet**}** | 0.15 ± 0.04 | 11.58 ± 0.16 | 33.42 ± 0.41 | 8.53 ± 0.22 | 8451 ± 542 | 11.2 : 88.8 | 5 |
| Mgobozeleni Estuary | Nov 2010 **{**wet**}** | 0.50 ± 0.07 | 0.71 ± 0.21 | 25.22 ± 0.68 | 8.87 ± 0.16 | 4141 ± 265 | 30.9 : 69.1 | 13.4 |
| Mgobozeleni Estuary | Nov 2009 **{**wet**}** | 0.7 ± 0.10 | 4.18 ± 1.44 | 22.05 ± 0.88 | 8.84 ± 0.31 | 37 ± 8 | 1.3 : 98.7 | 1.4 |
| Lake Sibaya | Nov 2010 **{**wet**}** | 0.7 ± 0.08 | 0.46 ± 0.10 | 28.48 ± 1.66 | 8.14 ± 0.22 | 7884 ± 526 | 42.1 : 57.9 | 4.3 |
| Lake Sibaya | Nov 2009 **{**wet**}** | 1.5 ± 0.10 | 0.18 ± 0.08 | 22.91 ± 0.15 | 8.54 ± 0.42 | 6045 ± 1021 | 31.6 : 68.4 | 3 |
| Lake Nhlange | Nov 2010 **{**wet**}** | 0.30 ± 0.07 | 3.03 ± 1.02 | 23.12 ± 0.26 | 8.87 ± 0.11 | 8213 ± 726 | 15 : 85 | 3.4 |
| Lake Nhlange | Nov 2009 **{**wet**}** | 0.30 ± 0.03 | 0.91 ± 1.22 | 20.61 ± 0.85 | 8.89 ± 0.57 | 7841 ± 502 | 51.4 : 48.6 | 2.7 |

Data are presented as averages ± SD unless otherwise indicated.

**^a^** Percentage proportion of juveniles (< 5.99 mm shell height) to adults (6 – 30 mm shell height).

**^b^** Average number of unborn juveniles in brood pouch per adult.
